# Unlocking the Potential of Computed Tomography-Guided Tracers in Pinpointing Lung Lesions during Surgery: A Collaborative Multi-Institutional Journey †

**DOI:** 10.3390/jcm13206041

**Published:** 2024-10-10

**Authors:** Rossella Potenza, Marco Andolfi, Andrea Dell’Amore, Marialuisa Lugaresi, Gabriella Roca, Leonardo Valentini, Chiara Catelli, Francesco Buia, Giampiero Dolci, Chiara Floridi, Riccardo Moretti, Claudia Colafigli, Majed Refai, Federico Rea, Francesco Puma, Niccolò Daddi

**Affiliations:** 1 Thoracic Surgery Unit, University of Perugia Medical School, 06129 Perugia, Italy; rossella.potenza1987@gmail.com (R.P.); francesco.puma@unipg.it (F.P.); 2 Thoracic Surgery Unit, AOU delle Marche, 60121 Ancona, Italy; majedit@yahoo.com; 3 Thoracic Surgery Unit, Department of Cardiac, Thoracic, Vascular Sciences, University of Padua, 06129 Padua, Italy; andrea.dellamore@unipd.it (A.D.); gabriella.roca@aopd.veneto.it (G.R.); chiara.catelli1992@gmail.com (C.C.); federico.rea@unipd.it (F.R.); 4 Department of Medicine and Surgery (DIMEC), University of Bologna, 40126 Bologna, Italy; marialuisa.lugaresi2@unibo.it; 5 Thoracic Surgery Unit, Alma Mater Studiorum—IRCSS Ospedaliero-Universitaria S. Orsola di Bologna, 40138 Bologna, Italy; leonardo.valentini4@studio.unibo.it (L.V.); giampiero.dolci@ospfe.it (G.D.); 6 Cardio-Thoracic-Radiology Unit, Department of Cardio-Thoracic-Vascular, IRCSS Ospedaliero-Universitaria S. Orsola di Bologna, 40138 Bologna, Italy; francesco.buia@aosp.bo.it; 7 Department of Radiological Sciences, Università Politecnica Marche, AOU delle Marche, 60121 Ancona, Italy; chiara.floridi@ospedaliriuniti.marche.it; 8 Department of Radiology, Santa Maria della Misericordia Hospital, 06129 Perugia, Italy; riccardo.moretti@ospedale.perugia.it (R.M.); claudia.colafigli@ospedale.perugia.it (C.C.)

**Keywords:** hook wire, microcoil, bioabsorbable hydrogel plug

## Abstract

**Background:** Multiple techniques exist for the preoperative localization of small, deeply located solid or subsolid pulmonary nodules to guide limited thoracoscopic resection. This study aims to conduct a multi-institutional comparison of three different tomography-guided tracers’ methods. **Methods:** A retrospective multicenter cross-sectional study was conducted. All patients suitable for CT-guided tracers with microcoil (GROUP1, n = 58), hook wire (GROUP2, n = 86), or bioabsorbable hydrogel plug (GROUP3, n = 33) were scheduled for video-assisted thoracoscopic wedge resection. Outcome variables: successful nodule localization, safety, and the feasibility of the tracers’ placement. A χ^2^ test or Fisher’s test for expected numbers less than five and a Kruskal–Wallis test were used to analyze the categorical and continuous variables, respectively. For the power calculations, we used G*Power version 3.1.9.6. **Results:** One hundred seventy-seven patients underwent the localization and resection of 177 nodules detected with three different CT-guided tracers. A significant difference was recorded for cancer history (*p* = 0.030), respiratory function, Charlson comorbidity index (*p* = 0.018), lesion type (*p* < 0.0001), distance from pleura surface (*p* < 0.0001), and time between preoperative CT-guided tracers and surgical procedures (*p* < 0.0001). Four post-procedural complications were recorded and in GROUP2, four cases of tracer dislocations occurred. Finally, hook wire group was associated with the shortest surgical time (93 min, *p* = 0.001). **Conclusions:** All methods were feasible and efficient, resulting in a 100% success rate for the microcoils and the bioabsorbable hydrogel plugs and a 94.2% success rate for the hook wires. Our results highlight the need to choose a technique that is less stressful for the patient and helps the surgeon by extending the approach to deep nodules and resecting over the course of several days from deployment.

## 1. Introduction

The use of low-dose computed tomography (CT) scans in high-risk patient screening programs and for long-term follow-up in cancer patients has led to an increasing number of detected pulmonary nodules [1].

Although bronchoscopic technologies can be considered the safest and most accurate tools for diagnosing both central and distal airways, they are often limited by nodule size, small tissue samples, and a challenging location for conventional approach [2]. Therefore, in the era of video-assisted thoracic surgery (VATS) and robotic-assisted thoracic surgery (RATS), radiologists’ guidelines propose limited resections for suspicious nodules, whether solid or subsolid, even without histologic proof of malignancy [3,4]. Indeed, VATS and RATS have both been proven to be safe and reliable for oncologic outcomes and are commonly practiced, offering several advantages such as reduced post-operative pain, hospital stay, and complications compared with the traditional open approach [5]. However, these minimally invasive approaches do not allow the digital palpation of the entire lung parenchyma, making it difficult to localize small and deep pulmonary nodules. Even more complex is the identification of ground glass opacities (GGOs), especially those with no solid components. In order to face this problem, various techniques for localizing small, deeply located subsolid nodules have been described, such as the preoperative percutaneous insertion of microcoils or hook wires or the percutaneous injection of bioabsorbable plugs, each with its own advantages and disadvantages [6].

In particular, microcoil placement has a low rate of pneumothorax/hemorrhage; it can be placed up to 2 days before the operation but requires intraoperative fluoroscopy, exposing both the patient and the operative team to radiation [6]. The injection of a bioabsorbable hydrogel plug is a valuable tool for reducing the incidence of post-biopsy pneumothorax, and due to the long absorption time, it eliminates the need for close cooperation between the radiological and the surgical procedure [6,7]. Finally, hook wires do not increase the radiation risk and do not require specialized equipment nor expertise but are associated with the highest rate of post-procedural pneumothorax, which may occur in up to 55% of patients; moreover, the surgical procedure needs to be scheduled on the same day as the CT-guided localization [6].

Therefore, many authors have tried to find the ideal tracer with a high accuracy rate, a low morbidity rate, minimal patient discomfort, applicability to all areas of the lung, no radiation exposure, and cost-effectiveness. However, to date, considering the few retrospective comparative studies published [6], an optimal method of preoperative localization for pulmonary nodules has not been established.

The current study aims to compare three different radiologic preoperative pulmonary nodule localization methods using CT guidance (microcoils, hook wires, and bioabsorbable hydrogel plugs) followed by VATS resection, analyzing the efficacy, safety, and feasibility of CT-guided tracer placement, in order to provide practical insights for clinical practice.

## 2. Materials and Methods

### 2.1. Ethical Statement

This retrospective, multicenter, cross-sectional study was approved by the Institutional Review Board on 13 September 2023, with the registration number 27582/23/ON. Since this study is retrospective in nature, individual informed consent was not required. Each patient had previously signed a general informed consent for scientific purposes upon admission to the hospital.

### 2.2. Study Population

This study involved a review of patients who underwent lung nodule localization with CT-guided tracers using a microcoil (GROUP1), hook wire (GROUP2), or bioabsorbable hydrogel plug (GROUP3), followed by subsequent VATS resection between January 2014 and December 2021.

All patients were scheduled to undergo VATS wedge resection (WR) at 4 Italian departments of thoracic surgery: Ancona (GROUP1), Perugia (GROUP2), Bologna (GROUP3), and Padua (GROUP2-3). The inclusion criteria were as follows:

1. Suspicious subsolid nodules;
2. Solid nodules measuring less than 2 cm and variably profoundly located.

Medical and radiological data were retrospectively reviewed. All marked nodules included in this study had a peripheral growth. On the contrary, patients with a central lung nodule did not require CT-guided tracer placement considering they directly underwent anatomic lung resection (segmentectomy or lobectomy) or resection through the traditional open approach.

Pre-procedure evaluations, which were comprehensive and thorough, included a detailed medical history, blood tests, cardiopulmonary assessment, total-body CT scans, and CT-PET.

All cases were discussed in a multidisciplinary meeting to evaluate the best diagnostic/therapeutic planning. In all patients, a trans-bronchial or percutaneous lung biopsy was unsuccessful or considered unfeasible due to the nodule position, size, or morphological aspects (i.e., pure GGOs) that would have put the procedure at high risk of false-negative results. In response, a proactive approach was taken, proposing CT-guided tracer insertion and surgery to avoid ineffective procedures and ensure patient safety.

When dealing with subpleural nodules with a GGO aspect, the decision to perform a surgical resection guided by the tracer was made, taking into consideration the nature of the lesion and the emphysematous radiological pattern of the lung that might have hindered intraoperative identification.

The available tracer helped to choose between three techniques. The expertise and skills of the dedicated interventional radiologists in each institution were utilized, using a tomography system with standardized protocols and safety radiation protection according to the European CE mark.

Post-procedural complications included pneumothorax, hemothorax, or other events requiring immediate treatment.

### 2.3. Preoperative Nodule Localization

#### 2.3.1. Microcoil

All microcoil positioning included in this study was performed in a CT suite equipped with a 64-detector CT scanner (LightSpeed VCT, GE Healthcare, Waukesha, WI, USA). Patients were positioned depending on the location of the lesion and the most appropriate access mode.

The radiologist delimitated the region of interest with a radiopaque grid. A chest CT scan was performed to confirm the location of the lesion and to plan the microcoil positioning. According to thoracic surgeons, the radiologist planned, on the CT images, the entry point on the skin plane and the target point close to the target nodule. In particular, the operator marked the entry point on the skin based on coordinates given by the intersection of grid markers and the CT level. When the needle-tip achieved the planned nodule margin, the stylet was removed from the needle, and one microcoil (2 mm × 10 mm, Boston Scientific, Watertown, MA, USA) was pushed into the needle by the stylet. Microcoils were always placed beyond the nodule in order to mark a sure free margin of depth for resection. Once the microcoil deployment was completed, a CT scan was performed to evaluate its position and possible complications. Finally, specific multiplanar images were reconstructed from the last CT acquisition for the surgeon’s evaluation and surgical approach planning.

The mean total dose–length product (DLP) radiation was 870 mGycm (SD ± 175).

#### 2.3.2. Hook Wire

The CT-guided hook wire was positioned on the same day as the planned surgery, 1 to 2 hours prior to surgery. The patient was placed on the CT scan table (Siemens Somatom Definition AS, Erlangen, Germany; GE Optima 64, Tokyo, Japan) in a position that provided the shortest access to the nodule. A grid of parallel marks was placed on the patient’s skin, and the first scan was performed. In collaboration with the radiology technician, the needle entry point on the skin was marked at the intersection between the CT gantry laser line at the reference slice and the correct line of the grid. The area was prepped and draped in the usual sterile fashion. Subsequently, subcutaneous and deep local anesthesia was administered via a 20 G needle along the intended path of the needle. A second scan was usually performed with the needle to verify the correct position. Then, a 10.7 cm long 20 G cannula was inserted towards the target, in a plane deeper than the nodule, ensuring that the stapler section line (including the entire hook wire) guarantees the complete removal of the lesion with a safe resection margin. A control scan was performed to check the position of the wire and to identify any possible complications. Finally, the skin was covered with sterile gauze, leaving the metal landmark at the center. The patient was placed on a bed in the same position in which the whole procedure was carried out and then transferred to the OR for surgery.

The mean total DLP radiation was 815 mGycm (SD ± 215).

#### 2.3.3. Bioabsorbable Hydrogel Plug

The procedures followed the same technique as described previously [6]. Procedures were performed by a staff of 3 interventional thoracic radiologists with 2–15 years of experience. Patients were positioned on the CT table based on the location of the lesion. A conventional chest CT scan with 2.5 mm slice thickness (Philips iCT SP 128, Best, The Netherlands) was conducted to visualize the lesion and the path for needle insertion. After entry site cleaning and local anesthesia administration, the operator advanced a 19-gauge coaxial needle step by step using CT images (with multiplanar reconstructions) to ensure accurate placement. After confirming the correct position of the needle, the sterile pouch was opened. This kit contained the deployment system with the biopsy tract plug, which the manufacturer had already preloaded. The BioSentry device (AngioDynamics, Queensbury, NY, USA) is a dehydrated polyethylene glycol hydrogel that expands as a solid cylinder (2.5 cm in length by 0.1 cm in diameter) when in contact with lung tissue. The coaxial introducer needle hub was prehydrated with saline, and the adapter was connected and secured. The deployment system was then mounted over the coaxial needle and adapter. The plunger was then pushed, advancing the plug to a predetermined depth depending on the distance between the skin and the pleura surface. After removing the needle and the deployment system, the plug expanded to fill the tract. Immediately after the procedure, a CT scan was performed to identify potential complications [6].

The mean total DLP radiation was 952 mGycm (SD ± 252).

### 2.4. Surgical Procedure

Due to the peripheral nodule localization, all patients initially were scheduled for VATS WR for diagnostic purposes.

The surgical procedure was limited to WR in cases of a history of cancer in another district/benign lesions, patients unfit for lobectomy, or possible multifocal lung cancer. Completion lobectomy plus systematic lymphadenectomy was performed if intraoperative diagnosis of non-small-cell lung cancer (NSCLC) was obtained.

**Figure 1:**
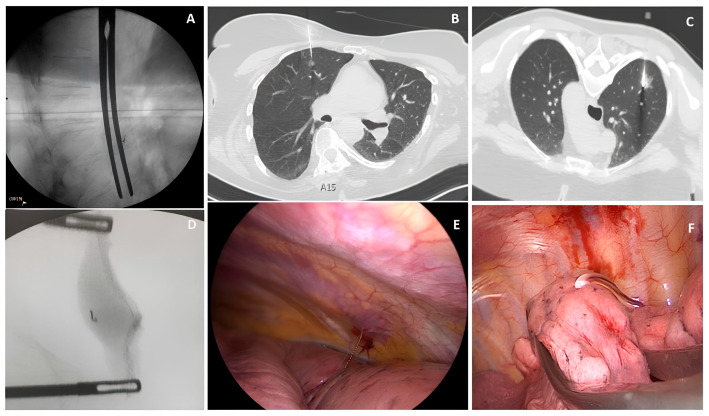
Tracers’ placement and intraoperative findings. (**A**) Intraoperative fluoroscopy finding before WR: microcoil above the forceps; (**B**) CT-guided hook wire insertion; (**C**) CT-guided hydrogel plug insertion; (**D**) intraoperative fluoroscopy after WR: microcoil included in the surgical specimen with wide free margins; (**E**) intraoperative finding of hook wire; (**F**) intraoperative finding of hydrogel plug.

**Figure 2:**
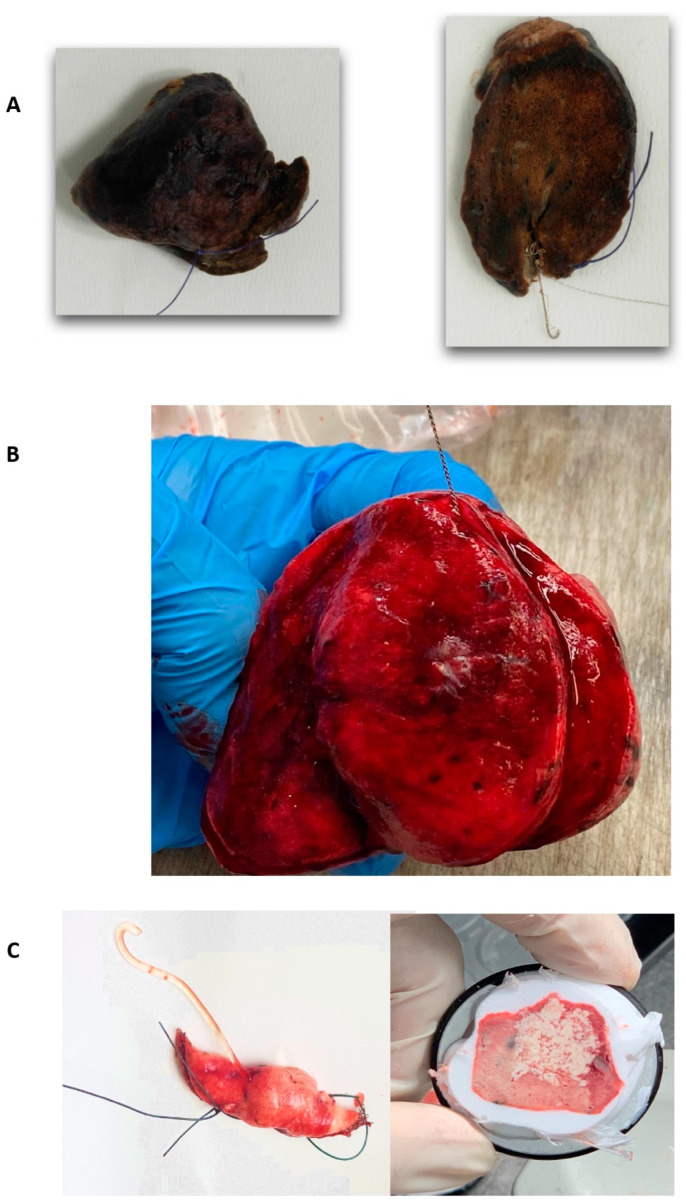
Surgical specimens. (**A**) Surgical specimen with microcoil; (**B**) surgical specimen with hook wire; (**C**) surgical specimen with hydrogel plug.

The surgical procedures were performed with the patient in a lateral position, using a triportal/biportal/uniportal VATS approach and double-lumen oro-tracheal intubation for single-lung ventilation under general anesthesia. Intraoperative fluoroscopy was used to locate the microcoil. At the end of the procedure, additional fluoroscopy was performed on the specimen to check for the presence of the microcoil (Figure 1A,D and Figure 2A).

In the case of hook wires, WR was performed as deep as possible, without the need for fluoroscopy or an intraoperative CT scan (Figure 1B,E and Figure 2B).

The plug tracer resection was performed under direct vision; if the plug was barely visible on the surface of the lung, an endoscopic linear ultrasound (ESAOTE LP 4-13, Esaote SpA, Genoa, Italy) was used (Figure 1C,F and Figure 2C).

Intraoperative frozen sections were always required to confirm the correct nodule removal, define its nature, and check the suture line in case of tumor.

### 2.5. Statistical Analysis

Post-procedural complications were defined as events caused by tracers’ deployment, requiring immediate bedside or surgical treatment. 

The outcomes were successful nodule localization, safety, and the feasibility of the tracers’ placement. Data are represented as the mean and range for continuous variables and n (%) for categorical variables. A χ^2^ test or Fisher’s test for expected numbers less than five and a Kruskal–Wallis test were used to analyze the categorical and continuous variables, respectively. For the power calculations, we used G*Power version 3.1.9.6.

## 3. Results

Over the period between January 2014 and December 2021, 177 patients underwent the preoperative localization of 177 undetermined lung nodules with the techniques mentioned above. There were 58 patients (27 males/31 females) in the microcoil group (GROUP1), 86 (43 males/43 females) in the hook wire group (GROUP2), and 33 (20 males/13 females) in the bioabsorbable hydrogel plug group (GROUP3). The details are shown in Table 1. The radiological characteristics of the lesions in GROUP1, 2, and 3, respectively, were as follows: GGO lesions were 10/58, 24/86, and 12/33; subsolid lesions were 0/58, 25/86, and 8/33; and solid lesions were 48/58, 37/86, and 13/33 (*p* < 0.0001 Kruskal–Wallis test, see Table 2). 

One hundred and seventy-seven nodules were localized and resected. No differences in the smoking history, sex, age, lesion size at the CT scan and at the pathological specimen, and post-surgical complications were detected (see Table 2). Moreover, no statistically significant correlation was found between the post-operative complications and comorbidity (*p* = 1.000) or Charlson index (*p* = 0.953). However, smokers developed more complications compared with previous smokers (*p* = 0.010), even if no difference was found between the two groups in terms of the Charlson comorbidity index (*p* = 0.360).

The mean time elapsed between the preoperative localization and surgical procedure was 30 h (4–96) for microcoils (GROUP1), 6 h (4–88) for hook wires (GROUP2), and 143 h (4–2592) for bioabsorbable plugs (GROUP3) (*p* < 0.0001 Kruskal–Wallis test). However, this delay did not impact on the post-procedural and post-operative complications. For GROUP2, the delay between marking and surgery was due to unexpected emergencies (i.e., lung transplant) in one institution (Padua) that prolonged the time for surgery planned on an elective day. For GROUP3, the surgery was conducted at almost four months after the CT-guided deployment in a GGO lesion due to the reluctance of the patient to undergo the surgical procedure. The GGO was resected and found to be an atypical adenomatous hyperplasia in the final pathological report.

During intraoperative thoracoscopic nodule localization, the use of microcoils and bioabsorbable hydrogel plugs resulted in a 100% success rate. However, in GROUP2, there were four cases of tracer dislocations. Despite this, thoracoscopic nodule resection was still possible because the puncture on the visceral pleural surface was visible. In addition, one more patient from GROUP2 required a salvage lobectomy due to an intraparenchymal hemorrhage following the tracer’s deployment, resulting in a success rate of 94.2%.

Four post-procedural complications required intervention: one pneumothorax in GROUP1 (1.72%); one intraparenchymal hemorrhage requiring a straightforward lobectomy in GROUP2 (1.16%); and one pneumothorax and one hemothorax in GROUP3 (6.06%). A significant difference was recorded for cancer history, respiratory function, Charlson comorbidity index, lesion type, distance from pleura surface (mean distance was 1.63 cm, 0.87 cm, and 1.55 cm in GROUP1, GROUP2, and GROUP3, respectively—*p* < 0.0001 Kruskal–Wallis test), and lymphadenectomy (11/58 in GROUP1; 27/86 in GROUP2; 21/33 in GROUP3—*p* < 0.0001). The mean CT-guided intervention time was 25 min (range, 20–30 min) for GROUP1, 30 min (range, 25–35 min) for GROUP2, and 28 min (range, 21–33) for GROUP3.

A significant difference in the length of surgical time was found (*p* = 0.001 Kruskal–Wallis test): 112 min (range 30–240), 93 (range 20–240), and 150 (range 15–355), respectively, for GROUP1, 2, and 3, including the average time needed by the pathologist for the frozen sections (30 min). This can be explained because in GROUP3 we had a higher percentage of anatomical resections with lymphadenectomies (48.48%) compared to the other two groups (18.9% GROUP1 and 23.2% GROUP2). No thoracotomy was required for the intraoperative identification of the nodules.

Histology revealed ninety-seven lung cancers (54.80%; eighty adenocarcinomas, ten squamous cell carcinomas, seven typical carcinoids), fifty-six metastases (31.6%), and twenty-four benign lesions (13.6%). The margins from all surgical specimens were free of disease.

Post hoc analysis revealed that this study had 0.8482739 power to detect a 0.25 effect size.

## 4. Discussion

Lung cancer screening programs for high-risk patients and CT follow-up in patients with a history of cancer have led to an increase in the detection of lung nodules [1]. Despite the vital help of radiologists’ guidelines, managing an indeterminate pulmonary nodule may be difficult. Sometimes, in the case of suspicious lesions, trans-bronchial or CT-guided biopsy may be limited by the nodules’ location or small size. In these cases, a limited VATS resection for definitive diagnosis/treatment may be suggested [3,4].

Although VATS has become common practice, problems arise with small, deeply located subsolid lesions. It has been reported that lesions presenting one of the following characteristics on CT scans may be difficult to localize during VATS: (1) a diameter measuring 10 mm or less, (2) nodule deep to the pleural surface, and (3) a subsolid component [8]. In a study by Suzuki et al., the risk of failure in detecting a nodule during VATS was 63% if the nodule was more than 5 mm deep to the pleural surface and less than 10 mm in size [9].

The ideal characteristics of an intraoperative localization device have been described: a high accuracy rate, a low morbidity rate, minimal patient discomfort, applicability to all areas of the lung, no radiation exposure, and cost-effectiveness. Accordingly, many techniques have been developed, including microcoil placement, hook wires, bioabsorbable hydrogel plugs, contrast media, radiotracers, injection dyes, a fluorescence tracer with near-infrared imaging, a CT fluoroscopy-guided injection of Cyanoacrylate, endobronchial ultrasound, and electromagnetic navigation bronchoscopy, each with advantages/limitations [10]. Microcoil placement has a low rate of pneumothorax/hemorrhage but requires intraoperative fluoroscopy, exposing both the patient and the operative team to radiation; migration with localization failure has also been described in up to 10% of patients. Moreover, they can be placed up to 2 days before the operation [6].

Contrast media are retained in the parenchyma for up to 3 months, eliminating the need for close cooperation between interventional radiology and the operative room (OR); inflammatory tissue reactions and embolisms have been reported [10].

CT-guided dye injection is a convenient method, but it quickly spreads into the surrounding parenchyma, making the identification of the nodule challenging, especially in patients with anthracotic pigmentation [9]. Intraoperative ultrasound is a non-invasive procedure that requires the complete collapse of the lung (which, in the case of emphysema, may be time-consuming), dedicated probes, and special skills, just like electronavigation bronchoscopy with dye injection, which is performed in the OR under general anesthesia [11,12].

The bioabsorbable hydrogel plug is a desiccated polyethylene glycol hydrogel that self-expands when in contact with lung tissue. This device was first described by Ahrar et al. as a valuable tool for reducing the incidence of post-biopsy pneumothorax [13], so the expected advantage, if used for the preoperative localization of lung nodules, is a reduction in the incidence of post-localization pneumothorax. Moreover, the plug is absorbed in 6 to 9 months, eliminating the need for close cooperation between the interventional radiology and the OR. In a study by Giunta et al., the technique was employed in three patients with good results for the intraoperative localization of small nodules/GGOs. All patients underwent successful nodule resection with clear intraoperative identification of the plug [7]. These data were subsequently confirmed in a series of 24 patients who underwent preoperative localization followed by VATS resection. In this series, only one patient (4%) developed a symptomatic pneumothorax; intraoperative nodule identification/resection was always achieved, even in the three patients (12.5%) in whom the dislodgment of the tracer occurred [14].

Hook wires do not increase the radiation risk and do not require specialized equipment nor expertise but are associated with a higher rate of dislodgement and pneumothorax than microcoils; moreover, the OR needs to be scheduled on the same day as the CT-guided localization. The reported complications are pneumothorax, parenchymal hemorrhage, hemoptysis, air embolism, and dislodgment.

Hook wire positioning has the highest rate of post-procedural pneumothorax, which may occur in up to 55% of patients. Several risk factors have been identified, such as the trans-fissural/trans-emphysema approach, localization of multiple nodules, and depth of insertion [15,16].

The reported incidence of dislodgment is 7.5% for hook wires [6], 0.6–7% for microcoils [17,18,19,20], and 12% for bioabsorbable hydrogel plugs [14]. In our experience, 4/86 patients (4.65%) belonging to GROUP2 experienced tracer dislodgment during VATS. As reported by Mullan, hook wire dislodgment can occur during three different moments: during the transportation of patients to the OR, during the deflation of the lung, and during the intraoperative manipulation of the lung [21]. It is possible that in these patients, a post-procedural and undetected pneumothorax developed, producing a pulling of the intraparenchymal portion of the hook wire and finally leading to dislodgment [22].

We reported a 94.2% location success rate with the hook wire and a 100% location success rate with the microcoil or bioabsorbable hydrogel plug. Our results align with the literature data for the microcoil and hook wire, although the latter yields a widely variable localization success rate, ranging from 93% to 99% [8,23,24,25,26]. In line with the literature, hook wire dislodgment did not interfere with nodule resection, which was still possible because the puncture site was visible on the pleural surface, allowing for the easy identification and resection of the nodules [15,16,26,27,28,29,30].

Moreover, analyzing the surgical implications of a rapid and accurate nodule identification, CT-guided tracers together with the recent advancements in radiological reconstruction software and 3D modeling, can help the surgeon for a more precise and faster bronco-vascular dissection, particularly during VATS segmentectomies [31]. Additionally, for small pure GGO nodules, and especially for patients with multiple lesions, a limited WR may be the appropriate therapeutic choice. In these cases, the use of a tracer is valuable for achieving a complete resection and for accurate pathological evaluation afterward.

Finally, CT-guided tracers could play a crucial role for future potential applications in the setting of virtual-assisted lung mapping (VAL-MAP) before thoracoscopic segmentectomies [32]. Indeed, due to the increased spread of anatomic segmentectomy for older patients with comorbidities and younger patients with early-stage lung cancer or GGO [33], CT-guided percutaneous procedures can guarantee an accurate evaluation of the target pulmonary lesion and, consequently, an adequate safety margins from the intersegmental plane.

Confirming this, a retrospective cohort study reported that nineteen of twenty-four patients undergoing thoracoscopic segmentectomy after VAL-MAP had preoperative CT-guided percutaneous nodule localization, fifteen with dye and microcoils and four with dye only, demonstrating a success rate of 70.3% (71/101 marking attempts) [34].

Our study has certain limitations, such as a small patient group and an uncontrolled comparison between the three methods, which could lead to selection bias. Hence, no causality can be established, and definitive conclusions cannot be drawn. Therefore, this study is exploratory, and prospective trials are necessary to confirm the results identified during our analyses.

In conclusion, all methods were feasible and effective, resulting in a 100% success rate for the microcoil and the bioabsorbable hydrogel plug and a 94.2% success rate for the hook wire, in intraoperative thoracoscopic nodule localization. This emphasizes the importance of selecting a technique that is less invasive for the patient and may expand the approach not only to deep nodules but also to their resection over several days from the initial deployment. In particular, the bioabsorbable plug has expanded the potential for intervention beyond the four-day period following deployment, a possibility not observed in the microcoil and hook wire groups.

## Figures and Tables

**Table 1:** Clinical details.

Variables	GROUP1 Microcoil	GROUP2 Hook Wire	GROUP3 Plug	*p*-Value
Age	66 (24–83)	61 (9–85)	63 (23–79)	0.058
Sex				0.425
Male	27/58	43/86	20/33	
Female	31/58	43/86	13/33	
Smoking history				0.507
Never	21/58	35/86	13/33	
Current	11/58	8/86	6/33	
Previous smoker	26/58	43/86	14/33	
Cancer history				0.030
None	16/58	38/86	13/33	
Lung	2/58	9/86	2/33	
Other	40/58	38/86	16/33	
Both	0/58	1/86	2/33	
Respiratory function, mean% (range)				
FEV1%	97 (33–134)	85 (38–118)	97 (33–134)	0.004
DLCO%	76 (33–137)	100 (62–139)	76 (18.49–119)	<0.0001
Charlson comorbidity index, mean (range)	6 (1–10)	5 (0–9)	5 (2–9)	0.018

FEV1: forced expiratory volume in the first second; DLCO: diffusing capacity of the lungs for Carbon Monoxide.

**Table 2:** Radiological, surgical, and pathological details.

Variables	GROUP1 Microcoil	GROUP2 Hook Wire	GROUP3 Plug	*p*-Value
Lesion type				<0.0001
GGO	10/58	24/86	12/33	
Subsolid	0/58	25/86	8/33	
Solid	48/58	37/86	13/33	
Lesion size at CT, mean cm (range)	1.23 (0.4–2.4)	1.25 (0.14–2.9)	1.38 (0.1–2.4)	0.200
Lesion size at specimen, mean cm (range)	1.05 (0.3–2.4)	1.13 (0.14–2.4)	1.13 (0.1–2.4)	0.782
Distance from pleura, mean cm (range)	1.63 (0–4)	0.87 (0–3.1)	1.55 (0.4–4.1)	<0.0001
Time between tracer positioning and surgical resection, mean hours (range)	30 (4–96)	6 (4–88)	143 (4–2592)	<0.0001
Post-procedural complications	1/58	1/86	2/33	n.d.
Surgical procedure upfront	-	1/86	-	0.071
WR	47/58	66/86	17/33	0.008
Segmentectomy	1/58 rS2	3/86 rS9-10 rS6 rS1-2	4/33 Lingula-sparing-lobectomy rS6 rS3 Lingula	0.008
Completion of lobectomy	10/58	17/86	12/33	0.008
Surgical time, mean minutes (range)	112 (30–240)	93 (20–240)	150 (15–355)	0.001
Lymphadenectomy	11	27	21	<0.0001
Post-surgical complications	6	8	2	0.785
Days of chest drain, mean (range)	3 (1–19)	3 (1–20)	3 (1–13)	0.244
Histology				
Primary lung neoplasms	28/58	51/86	18/33	0.595
Secondary lung neoplasms	23/58	23/86	10/33	0.595
Not malignant lesions	7/58	12/86	5/33	0.595

GGO: ground glass opacity; CT: computed-tomography; WR: wedge resection.

## Data Availability

The data underlying this article will be shared on reasonable request to the corresponding author.

## References

[1] Mazzone P.J., Silvestri G.A., Souter L.H., Caverly T.J., Kanne J.P., Katki H.A., Wiener R.S., Detterbeck F.C. (2021). Screening for Lung Cancer: CHEST Guideline and Expert Panel Report. Chest.

[2] Andolfi M., Potenza R., Capozzi R., Liparulo V., Puma F., Yasufuku K. (2016). The role of bronchoscopy in the diagnosis of early lung cancer: A review. J. Thorac. Dis..

[3] MacMahon H., Naidich D.P., Goo J.M., Lee K.S., Leung A.N., Mayo J.R., Mehta A.C., Ohno Y., Powell C.A., Prokop M. (2017). Guidelines for Management of Incidental Pulmonary Nodules Detected on CT Images: From the Fleischner Society 2017. Radiology.

[4] Callister M.E., Baldwin D.R., Akram A.R., Barnard S., Cane P., Draffan J., Franks K., Gleeseon F., Graham R., Malhotra P. (2015). British Thoracic Society guidelines for the investigation and management of pulmonary nodules. Thorax.

[5] Hernandez-Vaquero D., Vigil-Escalera C., Pérez-Méndez I., Gutierrez A., Avanzas P., Wei Y., Diaz R., Silva J., Moris C., Pscual I. (2021). Survival After Thoracoscopic Surgery or Open Lobectomy: Systematic Review and Meta-Analysis. Ann. Thorac. Surg..

[6] Park C.H., Han K., Hur J., Lee S.M., Lee J.W., Hwang S.H., Seo J.S., Lee K.H., Kwon W., Kim T.H. (2017). Comparative Effectiveness and Safety of Preoperative Lung Localization for Pulmonary Nodules: A Systematic Review and Meta-analysis. Chest.

[7] Giunta D., Daddi N., Dolci G., Campisi A., Congiu S., Buia F., Bagni A., Dell’Amore A. (2019). A new image-guided technique for intraoperative localization of lung small solid nodules or ground-glass opacities with a self-expanding tract sealant device: A preliminary experience. Interact. Cardiovasc. Thorac. Surg..

[8] Xu X., Yao Y., Shen Y., Zhang P., Zhou J. (2015). Clinical Analysis of Percutaneous Computed Tomography-Guided Hook Wire Localization of 168 Small Pulmonary Nodules. Ann. Thorac. Surg..

[9] Suzuki K., Nagai K., Yoshida J., Ohmatsu H., Takahashi K., Nishimura M., Nishiwaki Y. (1999). Video-assisted thoracoscopic surgery for small indeterminate pulmonary nodules: Indications for preoperative marking. Chest.

[10] Cornella K.N., Repper D.C., Palafox B.A., Razavi M.K., Loh C.T., Markle K.M., Openshaw L.E. (2021). A Surgeon’s Guide for Various Lung Nodule Localization Techniques and the Newest Technologies. Innovations.

[11] Santambrogio R., Montorsi M., Bianchi P., Mantovani A., Ghelma F., Mezzetti M. (1999). Intraoperative ultrasound during thoracoscopic procedures for solitary pulmonary nodules. Ann. Thorac. Surg..

[12] Awais O., Reidy M.R., Mehta K., Bianco V., Gooding W.E., Schuchert M.J., Luketich J.D., Pennathur A. (2016). Electromagnetic Navigation Bronchoscopy-Guided Dye Marking for Thoracoscopic Resection of Pulmonary Nodules. Ann. Thorac. Surg..

[13] Ahrar J.U., Gupta S., Ensor J.E., Mahvash A., Sabir S.H., Steele J.R., McRae S.E., Avritscher R., Huang S.Y., Odisio B.C. (2017). Efficacy of a Self-expanding Tract Sealant Device in the Reduction of Pneumothorax and Chest Tube Placement Rates After Percutaneous Lung Biopsy: A Matched Controlled Study Using Propensity Score Analysis. Cardiovasc. Intervent. Radiol..

[14] Imperatori A., Fontana F., Dominioni L., Piacentino F., Macchi E., Castiglioni M., Desio M., Cattoni M., Nardecchia E., Rotolo N. (2019). Video-assisted thoracoscopic resection of lung nodules localized with a hydrogel plug. Interact. Cardiovasc. Thorac. Surg..

[15] Dendo S., Kanazawa S., Ando A., Hyodo T., Kouno Y., Yasui K., Minura H., Akaki S., Kuroda M., Shimizu N. (2002). Preoperative localization of small pulmonary lesions with a short hook wire and suture system: Experience with 168 procedures. Radiology.

[16] Liu J., Liang C., Wang X., Sun M., Kang L. (2021). A computed tomography-based nomogram to predict pneumothorax caused by preoperative localization of ground glass nodules using hook wire. Br. J. Radiol..

[17] Ng C.S., Hui J.W., Wong R.H. (2013). Minimizing single-port access in video-assisted wedge resection, with a hookwire. Asian Cardiovasc. Thorac. Ann..

[18] Iguchi T., Hiraki T., Gobara H., Fujiwara H., Matsui Y., Miyoshi S., Kanazawa S. (2016). CT fluoroscopy-guided preoperative short hook wire placement for small pulmonary lesions: Safety evaluation and identification of risk factors for pneumothorax. Eur. Radiol..

[19] Lizza N., Eucher P., Haxhe J.P., De Wispelaere J.F., Johnson P.M., Delaunois L. (2001). Thoracoscopic resection of pulmonary nodules after computed tomographic-guided coil labeling. Ann. Thorac. Surg..

[20] Mayo J.R., Clifton J.C., Powell T.I., English J.C., Evans K.G., Yee J., McWilliams A.M., Lam S.C., Finley R.J. (2009). Lung nodules: CT-guided placement of microcoils to direct video-assisted thoracoscopic surgical resection. Radiology.

[21] Mullan B.F., Stanford W., Barnhart W., Galvin J.R. (1999). Lung nodules: Improved wire for CT-guided localization. Radiology.

[22] Hu L., Gao J., Chen C., Zhi X., Liu H., Hong N. (2019). Comparison between the application of microcoil and hookwire for localizing pulmonary nodules. Eur. Radiol..

[23] Refai M., Andolfi M., Barbisan F., Roncon A., Guiducci G.M., Xiumé F., Salati M., Tiberi M., Giovagnoni A., Paci E. (2020). Computed tomography-guided microcoil placement for localizing small pulmonary nodules before uniportal video-assisted thoracoscopic resection. Radiol. Med..

[24] Miyoshi K., Toyooka S., Gobara H., Oto T., Mimura H., Sano Y., Kanazawa S., Date H. (2009). Clinical outcomes of short hook wire and suture marking system in thoracoscopic resection for pulmonary nodules. Eur. J. Cardiothorac. Surg..

[25] Ichinose J., Kohno T., Fujimori S., Harano T., Suzuki S. (2013). Efficacy and complications of computed tomography-guided hook wire localization. Ann. Thorac. Surg..

[26] Hanauer M., Perentes J.Y., Krueger T., Ris H.B., Bize P., Schmidt S., Gonzalez M. (2016). Pre-operative localization of solitary pulmonary nodules with computed tomography-guided hook wire: Report of 181 patients. J. Cardiothorac. Surg..

[27] Yao F., Wang J., Yao J., Xu L., Wang J., Gao L. (2018). Reevaluation of the efficacy of preoperative computed tomography-guided hook wire localization: A retrospective analysis. Int. J. Surg..

[28] Park J.B., Lee S.A., Lee W.S., Kim Y.H., Song I., Lee J.G., Hwang J.J. (2019). Computed tomography-guided percutaneous hook wire localization of pulmonary nodular lesions before video-assisted thoracoscopic surgery: Highlighting technical aspects. Ann. Thorac. Med..

[29] Lin M.W., Chen J.S. (2016). Image-guided techniques for localizing pulmonary nodules in thoracoscopic surgery. J. Thorac. Dis..

[30] Tian Y., Wang C., Yue W., Lu M., Tian H. (2020). Comparison of computed tomographic imaging-guided hook wire localization and electromagnetic navigation bronchoscope localization in the resection of pulmonary nodules: A retrospective cohort study. Sci. Rep..

[31] Gossot D., Potenza R., Ojanguren A., Seguin-Givelet A. (2019). How to improve the precision of closed chest sublobar resections. Precis. Cancer. Med..

[32] Andolfi M., Potenza R., Seguin-Givelet A., Gossot D. (2020). Identification of the intersegmental plane during thoracoscopic segmentectomy: State of the art. Interact. Cardiovasc. Thorac. Surg..

[33] Cao C., D’Amico T., Demmy T., Dunning J., Gossot D., Hansen H., He J., Jheon S., Petersen R.H., Sihoe A. (2016). Less is more: A shift in the surgical approach to non-small-cell lung cancer. Lancet Respir. Med..

[34] Yang S.M., Lin C.K., Chen L.W., Chen Y.C., Huang H.C., Ko H.J., Chen C.M., Sato M. (2019). Combined virtual-assisted lung mapping (VAL-MAP) with CT-guided localization in thoracoscopic pulmonary segmentectomy. Asian J. Surg..

